# The genetic landscape of dural marginal zone lymphomas

**DOI:** 10.18632/oncotarget.9678

**Published:** 2016-05-27

**Authors:** Karthik A. Ganapathi, Vaidehi Jobanputra, Fabio Iwamoto, Preti Jain, Jinli Chen, Luciano Cascione, Odelia Nahum, Brynn Levy, Yi Xie, Pallavi Khattar, Daniela Hoehn, Francesco Bertoni, Vundavalli V. Murty, Stefania Pittaluga, Elaine S. Jaffe, Bachir Alobeid, Mahesh M. Mansukhani, Govind Bhagat

**Affiliations:** ^1^ Department of Pathology and Cell Biology, Columbia University Medical Center, New York, NY, USA; ^2^ Department of Neurology, Columbia University Medical Center, New York, NY, USA; ^3^ Institute of Oncology Research and Oncology Institute of Southern Switzerland, Bellinzona, Switzerland; ^4^ Hematopathology Section, Laboratory of Pathology, National Cancer Institute, Bethesda, MD, USA; ^5^ Department of Pathology, New York Medical College, Valhalla, NY, USA

**Keywords:** dural marginal zone lymphoma, genome, mutations, TNFAIP3, NOTCH2

## Abstract

The dura is a rare site of involvement by marginal zone lymphoma (MZL) and the biology of dural MZL is not well understood. We performed genome-wide DNA copy number and targeted mutational analysis of 14 dural MZL to determine the genetic landscape of this entity. Monoallelic and biallelic inactivation of *TNFAIP3* by mutation (n=5) or loss (n=1) was observed in 6/9 (67%) dural MZL exhibiting plasmacytic differentiation, including 3 IgG4+ cases. In contrast, activating *NOTCH2* mutations were detected in 4/5 (80%) dural MZL displaying variable monocytoid morphology. Inactivating *TBL1XR1* mutations were identified in all *NOTCH2* mutated cases. Recurrent mutations in *KLHL6* (n=2) and *MLL2* (n=2) were also detected. Gains at 6p25.3 (n=2) and losses at 1p36.32 (n=3) were common chromosomal imbalances, with loss of heterozygosity (LOH) of these loci observed in a subset of cases. Translocations involving the *IGH* or *MALT1* genes were not identified. Our results indicate genetic similarities between dural MZL and other MZL subtypes. However, recurrent and mutually exclusive genetic alterations of *TNFAIP3* and *NOTCH2* appear to be associated with distinct disease phenotypes in dural MZL.

## INTRODUCTION

Diffuse large B-cell lymphoma is the most common subtype of B-cell non-Hodgkin lymphoma (B-NHL) occurring in the central nervous system (CNS) [1]. In contrast, the meninges are the most common primary (or secondary) site of involvement by low grade B-NHL [2]. The dura is a recognized but uncommon, primary site of occurrence of marginal zone lymphoma (MZL) and rare cases of intra-parenchymal MZLs, occurring in a perivascular location, have also been described [3, 4]. A female predilection has been documented for dural MZLs, and since they often present as solitary masses, they can mimic meningioma's clinically and on imaging studies [5–7]. Most patients are cured following excision and local adjuvant therapy but systemic recurrences have occasionally been reported [8]. Based on their morphologic and phenotypic features, primary dural MZLs are considered similar to mucosa-associated lymphoid tissue (MALT) lymphomas [5, 9]. Intriguingly, a high proportion of dural MZLs (up to 47%) show a prominent, light chain-restricted plasmacytic component, with a subset displaying clonal IgG4+ plasma cells in the absence of an IgG4-related disorder [6, 7].

Data regarding the chromosomal aberrations underlying dural MZLs are limited. Trisomy 3 has been reported as a recurrent cytogenetic abnormality in up to 50% of cases [6, 7]. The genomic and mutational landscape of dural MZLs, however, remains unexplored and the relationship of dural MZL with other MZL subtypes has not been defined [10–12]. In this report, we describe the results of genome-wide DNA copy number and targeted mutational analyses of 14 dural MZL. We detected recurrent and apparently mutually exclusive alterations in two genes critical for normal marginal zone B-cell development and maintenance or homeostasis, *NOTCH2* and *TNFAIP3*, which appear to be associated with distinct disease phenotypes, as well as a variety of additional genomic aberrations in common with other MZL subtypes.

## RESULTS AND DISCUSSION

All fourteen patients were adults (age range 33-62, median 46.5) and there was a marked female predominance (12 females, 2 males). None had a prior history of any hematological malignancy. Imaging studies showed solitary dural-based mass lesions (13 cranial, 1 spinal). All patients underwent surgical resection and 8/14 (57%) also received radiation (n=5) and/or chemotherapy (n=4). Ten of 11 (90%) patients with follow up data are alive and disease-free (follow up 12-120 months, median 29 months) and 1 patient died of a disease-unrelated cause (Table 1).

**Table 1:** Clinical features of dural MZL patients

Case No.	Age	Sex	Symptoms	Site	Imaging	Therapy	Outcome	Follow up duration (months)
1	62	F	Headaches, right sided discoordination	Cranial Dura	Solitary mass	RTX, CTX	Dead- Unrelated cause	29
2	48	F	Left jaw numbness, left-sided auditory defects	Cranial Dura	Solitary mass	Surgery	Alive- NED	77
3	33	F	Headaches, left sided weakness, seizures	Cranial Dura	Solitary mass	RTX	Alive- NED	26
4	57	F	Headache	Cranial Dura/Parotid	Solitary mass	Surgery, CTX	Alive-NED	20
5	45	F	Back pain and spasms	Spinal Dura	Solitary mass	N/A	Alive- NED	120
6	42	F	Headaches	Cranial Dura	Solitary mass	RTX	Alive- NED	29
7	60	F	N/A	Cranial Dura	Solitary mass	RTX	Alive- NED	22
8	34	F	Seizures	Cranial Dura	Solitary mass	CTX	Alive- NED	12
9	62	F	Headaches, right-sided numbness	Cranial Dura	Solitary mass	CTX	Alive-NED	N/A
10	50	F	N/A	Cranial Dura	Solitary mass	N/A	N/A	N/A
11	53	M	N/A	Cranial Dura	Solitary mass	Surgery	Alive-NED	204
12	43	F	Headache, syncope and neurologic deficits	Cranial Dura	Solitary mass	RTX	Alive-NED	108
13	42	F	Seizures	Cranial Dura	Solitary mass	N/A	N/A	N/A
14	37	M	N/A	Cranial Dura	Solitary mass	N/A	N/A	N/A

F: female; M: male; N/A: not available; RTX: radiation therapy; CTX: chemotherapy; NED: no evidence of disease

**Figure 1:**
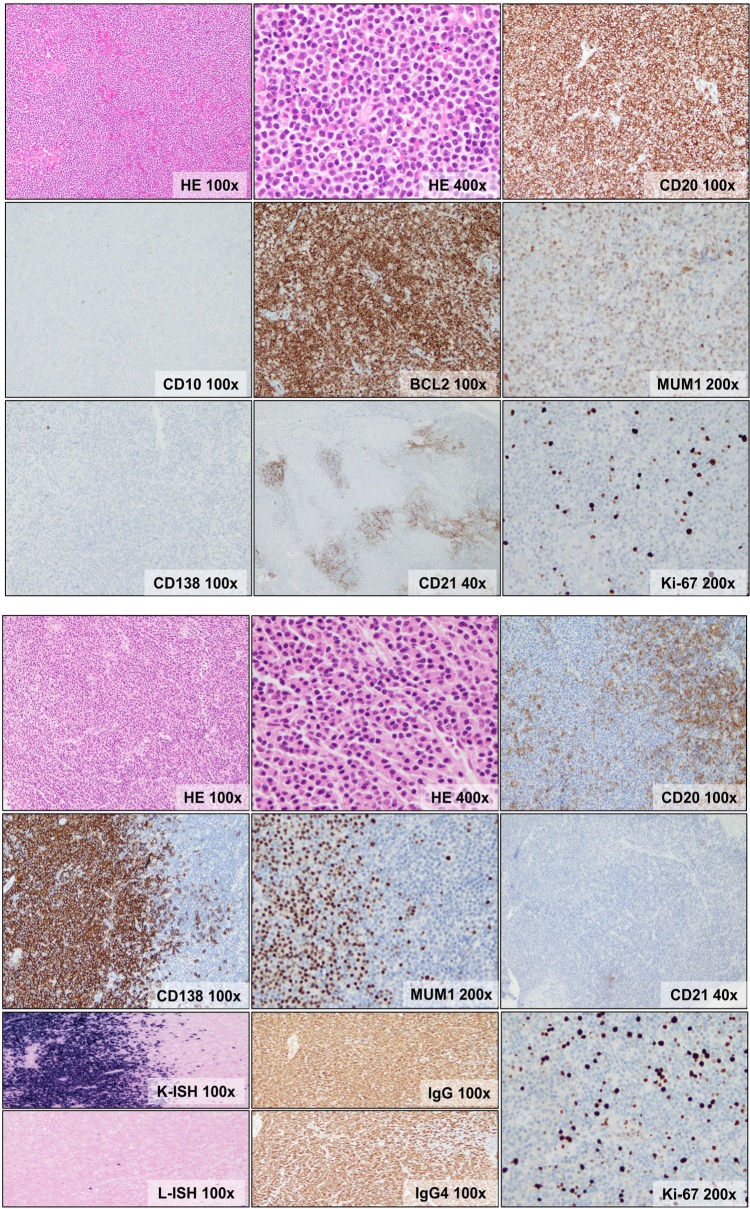
Morphologic and immunophenotypic features of dural MZL **A**. Dense, vaguely nodular lymphocytic infiltrate composed of atypical, small lymphocytes exhibiting irregular nuclei, fine chromatin, indistinct nucleoli and abundant pale cytoplasm (monocytoid appearance). The cells are positive for CD20, BCL2, MUM1/IRF4(weak), negative for CD138 and have a low proliferation index (Ki-67 <5%). CD138 shows rare plasma cells. CD21 shows disrupted follicular dendritic cell meshworks. **B**. Dense vaguely nodular lymphocytic and plasmacytic infiltrate composed of small lymphocytes, some exhibiting plasmacytoid features, and plasma cells. The lymphocytes express CD20, while the plasma cells express CD138 and MUM1/IRF4(bright) and they show kappa light chain restriction and IgG4 expression. CD21 shows rare, small and fragmented follicular dendritic cell meshworks. Ki-67 staining shows a low proliferation index (<10%).

Five cases exhibited variable monocytoid cytomorphology and nine cases showed extensive plasmacytic differentiation, with 6/9 cases (67%) displaying clonal IgG4+ plasma cells (Figure 1, Table 2). None of the latter had a history of sclerosing pachymeningitis (meningeal IgG4-related disease) or systemic IgG4-related disease before or after the diagnosis of lymphoma, supporting the notion that a subset of MZL represent localized and *de novo* IgG4-positive lymphoproliferations [7, 13].

**Table 2:** Histopathologic, cytogenetic and molecular features of dural MZL

Case No	Plasma cells	CD20	CD79a	MUM1/IRF4	CD138	K/L	IgG4	Ki-67	Karyotype	FISH	IGH PCR
1	−	+	+	−	−	Poly	−	5-10%	N/A	IGH=NR, 3 copies of MALT1	Clonal
2	−	+	+	±	−	Poly	−	5-10%	N/A	IGH=NR, MALT1=NR	Clonal
3	−	+	+	−	−	Poly	−	5-10%	Failure	IGH=NR, BCL6=NR, MALT1=NR	Clonal
4	−	+	+	−	−	Poly	−	10-20%	Failure	IGH=NR, BCL6=NR, MALT1=NR	Clonal
5	−	+	N/A	N/A	N/A	Poly	−	N/A	Failure	IGH=NR, MALT1=NR	Polyclonal
6	+	+	+	+	+	K	+	5-10%	N/A	IGH=NR, BCL6=NR, MALT1=NR	Clonal
7	+	+	+	+	+	K	+	10-20%	N/A	MALT1=NR	Clonal
8	+	+	+	+	+	K	−	40-50%	Normal	IGH=NR, BCL6=NR, MALT1=NR	Clonal
9	+	+	+	+	N/A	K	−	5-10%	N/A	IGH=NR, BCL6=NR, MALT1=NR	Clonal
10	+	+	+	+	N/A	K	+	20-50%	N/A	BCL6=NR	Clonal
11	+	+	+	+	N/A	K	+	N/A	Normal	IGH=NR, BCL6=NR, MALT1=NR	Clonal
12	+	+	N/A	+	N/A	L	+	N/A	Normal	IGH=NR, BCL6=NR, MALT1=NR	Clonal
13	+	+	N/A	+	N/A	L	+	N/A	N/A	IGH=NR, BCL6=NR, MALT1=NR	Failure
14	+	+	N/A	±	N/A	K	−	5-10%	Normal	BCL6=NR	Clonal

N/A: not available; Poly: polytypic; K: kappa light chain restriction; L: lambda light chain restriction; NR: not rearranged

G-band chromosome analysis showed normal karyotypes in 4 cases and it failed in 3 cases. Interphase FISH analysis using *IGH*, *MALT1* and *BCL6* probes showed no rearrangements but an additional copy of *MALT1* was noted as a subclonal change in one MZL (case 1). PCR analysis for immunoglobulin heavy chain (*IGH*) gene rearrangement showed clonal products in 12/13 (92%) evaluable cases (Table 2).

Targeted next generation sequencing analysis of 465 cancer-associated genes (Supplementary Table 1), with an average of 684-fold coverage and >10X coverage for 99% of the coding regions (Supplementary Table 2), revealed somatic mutations in all 11 cases (100%) with informative results; sequencing failed in 3 MZLs (cases 12-14).

**Figure 2:**
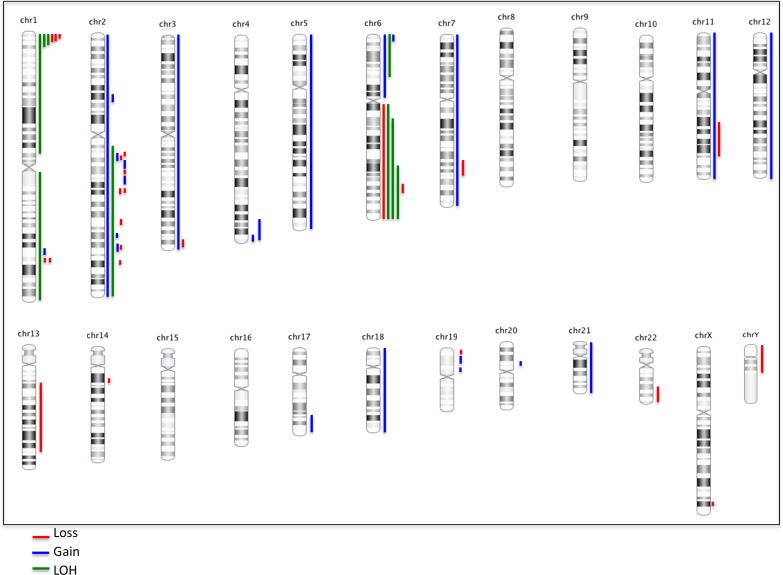
Summary ideogram showing genomic alterations in dural MZL Blue indicates gains, red indicates losses and green indicates loss of heterozygosity (LOH).

Sequencing and whole genome DNA copy number analysis showed inactivation of *TNFAIP3* in 6/9 (67%) cases exhibiting plasmacytic differentiation (Figure 2, Table 3). Loss of function mutations of *TNFAIP3* were identified in 5/9 (56%) cases, including two novel variants (Supplementary Table 3). Concomitant loss of heterozygosity (LOH) at 6q23 was noted in 2 cases, indicating bi-allelic *TNFAIP3* inactivation. Additionally, loss of 6q23 involving the *TNFAIP3* locus and LOH in this region were seen in one case each (1/9, 11%); poor DNA quality precluded assessment of mutations in these cases (Supplementary Table 4).

**Table 3:** Genetic abnormalities in the two morphologic variants of dural MZL

Case No	1	2	3	4	5	6	7	8	9	10	11	12	13	14
PC	−	−	−	−	−	+	+	+	+	+	+	+	+	+
IgG4	−	−	−	−	−	+	+	−	−	+	+	+	+	−
***NOTCH2***	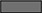	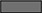	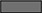	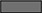										
	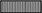	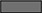												
***TNFAIP3***						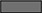	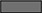	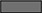	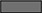	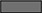				
								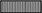		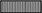			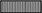	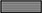
***TBL1XR1***	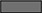	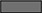	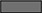	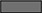										
													
***KLHL6***						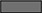					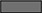			
													
***MLL2/KMT2D***		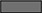						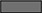						
													
***CARD11***											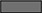			
													
***RHOA***										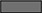				
													
***MLL/KMT2A***				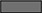										
													
***TNFRSF14***	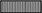	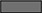	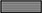		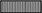	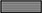				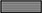		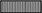		
													
***PTPRC***											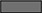			
													

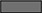
 **Mutation** 
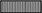
 **LOH** 
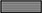
 **Deletion**

Columns represent individual cases and the rows represent genes (two rows for each gene represent the two alleles). PC: plasmacytic differentiation

TNFAIP3 (also known as A20) is a negative regulator of NF-κB signaling [14, 15]. B-cell specific deletion of *TNFAIP3* in mice results in mislocalization of marginal zone B-cells and defective antigen-induced B-cell maturation [16]. TNFAIP3-deficient B-cells are hyper-reactive to antigen stimulation, leading to enhanced proliferation and survival. Mice with B-cells lacking TNFAIP3 also demonstrate plasma cell hyperplasia and chronic inflammation, and they develop autoimmune disorders upon aging [16]. Recurrent inactivating mutations and/or genomic loss of *TNFAIP3* have been described in Hodgkin and non-Hodgkin lymphomas, including diffuse large B-cell lymphoma (DLBCL) [17, 18], extranodal MZL and nodal MZL [10, 11, 19]. However, an association with plasmacytic differentiation has not been reported for any type of B-NHL harboring this genetic alteration.

Activating *NOTCH2* mutations were identified in 4/5 (80%) cases manifesting variable monocytoid features, including three novel variants (Table 3, Supplementary Table 3). Bi-allelic *NOTCH2* aberrations were identified in two cases; bi-allelic mutations in one and a mutation accompanied by LOH at 1p11, containing the *NOTCH2* locus, in another. *NOTCH2* mutations were either located in the transactivation domain (TAD) or the proline/glutamate/serine/threonine-rich (PEST) domain, resulting in deletion of protein degradation motifs that regulate protein stability [20].

NOTCH2 is indispensable for marginal zone B-cell development and maintenance [21]. Targeted deletion of *NOTCH2* in murine B-cells results in the complete absence of marginal zone B-cells and their precursors i.e. transitional T2 B-cells [22]. Conversely, constitutively active NOTCH2 signaling in murine B-cells leads to an expansion of marginal zone B-cells at the expense of follicular B-cells. However, mice with constitutive NOTCH2 expression do not develop B-cell lymphoma, suggesting that sustained NOTCH2 signaling alone is insufficient for B-cell lymphomagenesis [23]. The majority of documented *NOTCH2* mutations in B-NHLs target the C-terminal transactivation (TAD) domain or the proline/glutamate/serine/threonine-rich (PEST) domain, resulting in increased protein stability and uncontrolled activation of the NOTCH2 and NF-κB pathways [24]. *NOTCH2* activating mutations have been identified in a variety of lymphomas, including splenic MZL, follicular lymphoma (FL) and DLBCL, and their presence is thought to predict an aggressive clinical course in certain B-NHLs [24–28]. Until now, *NOTCH2* mutations have not been described in non-splenic MZL.

Of note, recurrent *TBL1XR1* mutations (4/11, 36%) were only seen in association with *NOTCH2* mutations (Table 3, Supplementary Table 3), which could indicate a co-operative role for these mutations in lymphomagenesis. TBL1XR1 is a transcriptional regulator of the Wnt/β-catenin and NF-κB pathways and recurrent mutations of this gene have previously been described in other subtypes of B-NHL, especially splenic MZL and primary CNS DLBCL [28, 29]. Other recurrently mutated genes in dural MZL included *KLHL6* (2/11, 18%) and *MLL2/KMT2D* (2/11, 18%) (Table 3). A role in B-cell receptor signaling has been proposed for KLHL6 and recurrent *KLHL6* mutations have been described in chronic lymphocytic leukemia [30]. MLL2/KMT2D plays an important role in chromatin remodeling and transcriptional regulation and loss of function *MLL2/KMT2D* mutations have been described in a variety of B-NHL, including splenic MZL [28, 31, 32]. Non-recurrent mutations of *CARD11, RHOA*, *MLL/KMT2A*, *TNFRSF14,* and *PTPRC* were also identified and the variant allele frequencies of some mutations suggested that they might represent secondary events (Supplementary Table 3).

**Figure 3:**
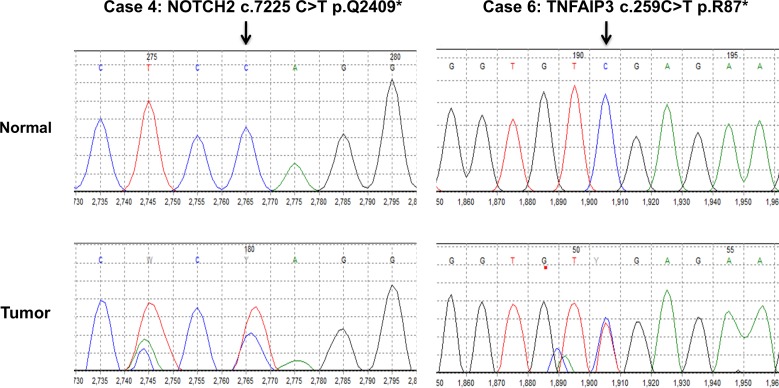
Sanger confirmation of *NOTCH2* and *TNFAIP3* mutations

The recurrent mutations described above were confirmed by Sanger sequencing (Supplementary Table 3, representative examples are shown in Figure 3).

Whole genome DNA copy number analysis showed chromosomal alterations in 12 of 13 cases (median - 4 per case, range 1-9), including recurrent aberrations involving 1p36.32 [loss, 3/13 (23%), LOH, 3/13 (23%)], with the minimal common region of aberration encompassing *TNFRSF14, PRDM16, RPL22* and *CAMTA1* genes, and 6p25.3 [gain, 2/13 (15%), LOH, 1/13 (8%)], encompassing the *IRF4* gene. Whole chromosome abnormalities were identified in 2/13 (15%) cases, including one case that showed gains of 3 and 18 (Figure 2, Supplementary Table 4), which were not observed by FISH analysis, potentially representing subclonal changes. The observed genomic changes are similar to those described previously in other MZL subtypes, although the frequencies appear to differ [10, 11]. Importantly, our findings suggest that in contrast to other MZL subtypes, aberrations of chromosomes 3 and 18 are infrequent in primary dural MZL [6, 7].

In summary, we report recurrent and apparently mutually exclusive genetic alterations of *TNFAIP3* and *NOTCH2* in dural MZL, in addition to novel and previously described mutations and genomic aberrations associated with other MZL subtypes. Contrary to observations in other B-NHL, these genetic aberrations do not appear to portend an aggressive course in dural MZL. Until now, specific genotype-phenotype correlations have not been reported for other MZL subtypes and our findings raise the possibility of at least two independent pathways leading to dysregulated NF-κB signaling in dural MZL that our associated with distinct disease phenotypes. However, larger studies with longer follow up are warranted to clarify the prognostic implications and functional consequences of the different genetic lesions discerned.

## MATERIALS AND METHODS

### Case selection

Fourteen primary dural MZL cases with adequate formalin-fixed paraffin-embedded (FFPE) tissue (>50% tumor) were identified in the archives of the Departments of Pathology, Columbia University Medical Center, New York (n=9) and the National Cancer Institute, Bethesda, Maryland (n=5). The latter were part of a previously published series [7]. Clinical, radiological and treatment data were reviewed for all cases. The study was performed using protocols approved by the institutional review boards of both institutions and in accordance with the principles of the Declaration of Helsinki.

### Morphologic and immunophenotypic evaluation

Hematoxylin and Eosin (H&E) stained FFPE tissue sections were reviewed to assess cyto-architectural features. Immunohistochemistry and in-situ hybridization was performed with the following panel of antibodies and probes; CD20 (clone MJ1); CD10 (clone 56C6); CD5 (clone 4C7); BCL6 (clone LN22); BCL2 (clone D5); CD21 (clone PA0171); kappa (clone ISH-5748A), lambda (clone ISH-5770A), all from Leica, IL, USA; CD79a (clone AP18); Cyclin D1 (clone SP4-R); CD138 (clone B-A38); CD21 (clone PA0171); IgG (clone 1210208A); IgG4 (clone 1123107A); Ki-67 (clone 30-9), all from Ventana, AZ, USA; and MUM-1 (clone MUM1p) from DAKO, CA, USA. Staining was performed with automated stainers (Ventana Benchmark Ultra and Leica Bond III) and visualized with the UltraView Universal and Bond polymer DAB detection kits according to the manufacturer's protocols.

### Cytogenetic analysis

Giemsa (G) banding and karyotype analysis was performed on metaphase preparations from fresh tumor specimens (n=7) after short-term (24 hr.) culture using standard techniques and karyotypes were described according to the International System for Human Cytogenetic Nomenclature [33].

Fluorescence in situ hybridization (FISH) analysis using *IGH* (n=11)*, MALT1* (n=12) and *BCL6* (n=10) break-apart probes (Abbott Laboratories, Abbott Park, Illinois, USA) was performed on FFPE tissue sections. FISH signals were scored on 200-500 interphase nuclei after counterstaining with DAPI using a Nikon Eclipse 600 microscope and captured with the Cytovision Imaging system (Applied Imaging, Santa Clara, CA).

### Immunoglobulin heavy chain (IGH) gene rearrangement analysis

DNA was extracted from FFPE tissue and fluorescent polymerase chain reaction (PCR) was performed using the BIOMED-2 primers, followed by capillary gel electrophoresis, to ascertain the presence of clonal *IGH* gene rearrangements, as described [34].

### Next generation sequencing

Targeted next generation sequencing was successful in 11 of 14 cases using a panel comprising 465 cancer-associated genes (Supplementary Table 1). Fifty to 250 ng of DNA, extracted using the Qiamp mini kit or the Qiamp FFPE kit (Qiagen, Germantown, USA) was fragmented to a median of 150-200bp, by sonication. Following end-repair and 3′ adenylation of the fragments, and ligation of double-stranded sequencing and indexing adaptors to ends, target capture and enrichment was performed with the Sure Select Hybrid Capture system (Agilent Technologies, Santa Clara, USA), using custom designed probes. Libraries were then quantified using qPCR, diluted to 2nM and pooled, prior to cluster generation and analysis on Illumina HiSeq2500, using Illumina TruSeq v3 chemistry (San Diego, USA) and 100bp paired-end reads (up to 9 indexed samples per run). Fastq files of reads where over 70% of reads were above Q30, were demultiplexed with CASAVA, and samples with at least 6Gb of data were used for mapping and variant calling using NextGene Software (Softgenetics, State College, USA), using the following criteria: 0 allowable ambiguous alignments, at least 90% of a read having to match the reference genome, software set to detect large indels, hiding the unmatched ends of reads, with at least 10% variant allelic fraction, and at least 3 variant reads to call a variant.

After annotation, the variants were cross referenced with those in the 1000 Genomes Project, OMIM, dbSNP, and the Exome Variant Server. Variants with an allele prevalence >1% in the 1000 genomes project were excluded. Common variants present in our departmental database of variants identified in prior constitutional exome analysis, non-pathogenic variants reported in dbSNP, and low quality calls were filtered out. The remaining variants were submitted for manual curation and variant prioritization with visual review of alignments. Synonymous variants and intronic variants greater than 2 bp from the coding sequence were excluded. Variants were manually cross-referenced with the Catalog of Somatic Variants in Cancer (COSMIC) and those that were not known “hot spot” mutations or had not been previously reported as potential driver variants were analyzed by PROVEAN and SIFT algorithms (Supplementary Table 2). Matched normal tissue samples (bone marrow aspirate) were available for two MZLs (cases 4 and 9). Variants present in the normal sample were excluded from analysis in these cases.

### Whole genome copy number and loss of heterozygosity (LOH) analysis

Genome-wide DNA copy number and LOH analysis was performed in 13 of 14 cases using the Affymetrix OncoScan FFPE assay (Affymetrix, CA, USA), which utilizes molecular inversion probe technology and is optimized to work on DNA derived from FFPE tissue samples [35].

Sample preparation, hybridization and scanning were performed according to the manufacturer's specifications. Analysis was performed using the Affymetrix Chromosome Analysis Suite 2.0 (ChAS) and Nexus Copy Number 7.5 software (Biodiscovery, Inc. CA, USA). All copy number alterations and regions of LOH recognized by the software were verified visually to determine erroneous calls and identify clonal or subclonal gains and losses not detected by the software. Analysis was restricted to gains and losses >1 Mb in length (Supplementary Table 4). Genomic alterations were reported based on the NCBI build 37 (hg19) of the human genome and cancer-associated genes were curated from the Cancer Gene Census (COSMIC v61 Release; http://www.sanger.ac.uk/genetics/CGP/Census/)

### Sanger Sequencing

Recurrent mutations deemed to be pathogenic were confirmed by Sanger sequencing according to standard methods. Briefly, PCR products obtained with specific primers flanking the mutation of interest were treated with exonuclease and shrimp alkaline phosphatase to remove remaining primers and dNTPs, and used as substrates for cycle sequencing using the BidDye Terminator Version 3.1 chemistry (Thermo Fisher, Springfield Township, USA), analyzed on an ABI 3130XL capillary sequencer (ThermoFisher, USA), and evaluated using Mutation Surveyor (Softgenetics, State College, USA).

## SUPPLEMENTARY TABLES








